# Sulforaphane exerts anti-angiogenesis effects against hepatocellular carcinoma through inhibition of STAT3/HIF-1α/VEGF signalling

**DOI:** 10.1038/s41598-017-12855-w

**Published:** 2017-10-04

**Authors:** Peng Liu, Samuel J. Atkinson, Sophia E. Akbareian, Zhigang Zhou, Andrea Munsterberg, Stephen D. Robinson, Yongping Bao

**Affiliations:** 1 Norwich Medical School, University of East Anglia, Norwich, Norfolk, United Kingdom; 2 School of Biological Sciences, University of East Anglia, Norwich, Norfolk, United Kingdom

## Abstract

Angiogenesis plays an important role in hepatocellular carcinoma (HCC), the inhibition of which is explored for cancer prevention and treatment. The dietary phytochemical sulforaphane (SFN) is known for its anti-cancer properties *in vitro* and *in vivo*; but until now, no study has focused on the role of SFN in HCC tumor angiogenesis. In the present study, *in vitro* cell models using a HCC cell line, HepG2, and human endothelial cells, HUVECs, as well as *ex vivo* and *in vivo* models have been used to investigate the anti-tumor and anti-angiogenic effect of SFN. The results showed that SFN decreased HUVEC cell viability, migration and tube formation, all of which are important steps in angiogenesis. More importantly, SFN markedly supressed HepG2-stimulated HUVEC migration, adhesion and tube formation; which may be due to its inhibition on STAT3/HIF-1α/VEGF signalling in HepG2 cells. In addition, SFN significantly reduced HepG2 tumor growth in a modified chick embryo chorioallantoic membrane (CAM) assay, associated with a decrease of HIF-1α and VEGF expression within tumors. Collectively, these findings provide new insights into the inhibitory effect of SFN on HCC tumor angiogenesis as well as tumor growth, and indicate that SFN has potential for the prevention and treatment of HCC.

## Introduction

Hepatocellular carcinoma (HCC) is the sixth most common cancer worldwide, and one of the most common causes of cancer death^1^. High mortality rates were reported in Asia and Africa, especially in less-developed regions^2^. Surgical resection or transplantation of liver offers the best prognosis among other limited treatments, but only 15% of HCC patients are suitable for surgical intervention after initial diagnosis. Non-surgical treatment is still in demand; however, most forms of HCC are highly radio- and chemo-resistant and desirable therapeutic outcome is often elusive in clinical cases^3^. Therefore, the development of new therapeutic agents for HCC patients is a priority.

Angiogenesis is believed to play a central role in the development and progression of HCC which is one of the most vascularized solid tumors possessing high micro-vessel density^4^. Angiogenesis is a complex process consisting of the release of angiogenic factors, binding of angiogenic factors to receptors on endothelial cells (ECs), EC activation, migration and proliferation, remodelling of the extracellular matrix (ECM) and tube formation^5^. Recent evidence suggests that tumor angiogenesis, including HCC, involves cascades of signalling between tumor cells and the host stroma microenvironment and leads to the formation of structurally and functionally abnormal vessels which contribute to tumor growth and metastasis^6^. All these steps provide opportunities to halt tumor growth and even promote tumor regression, thus angiogenesis pathways represent an attractive therapeutic target for HCC. Sorafenib, a multi-kinase inhibitor targeting amongst others VEGFR1, VEGFR2, and VEGFR3, was approved in 2008 for patients with advanced HCC. Many more antiangiogenic agents have reached advanced phases of development for the treatment of HCC^7^. However, the therapeutic efficacy of these agents proved to be limited because of acquired drug resistance, serious toxic side effects and high cost^3,6,8^. Therefore, the identification of alternative agents targeting angiogenesis is considered an important strategy both for HCC prevention and treatment.

Sulforaphane ([1-isothioyanato-4-(methyl-sulfinyl)butane], SFN) can be found at high levels in cruciferous vegetables such as broccoli and cauliflower^9^, and has been reported to exert a variety of bio-active effects including anti-oxidation, anti-inflammation, cytotoxicity and cytoprotection. Furthermore, *in vitro* and *in vivo* studies have revealed that SFN affects many stages of cancer development: it modulates the initiation phase of cancer by inhibiting phase I enzymes and inducing phase II enzymes; the promotion phase by inducing apoptosis, autophagy and cell-cycle inhibition; and the progression phase by inhibiting EMT, angiogenesis and metastasis. It showed anti-cancer effect on skin-, blood-, colon-, breast-, prostate-, pancreatic and other cancers^10,11^. In the case of HCC, SFN has shown chemopreventive^12–14^ and chemotherapeutic effects. It was found to decrease cell viability, telomerase activity^15^, and to induce apoptosis in hepatocellular carcinoma cells^16,17^ as well as in an orthotopic xenograft tumor model of HCC^18^. SFN also amplified tumor necrosis factor-related apoptosis-inducing ligand (TRAIL) induced apoptotic signalling in TRAIL-resistant hepatoma cells^19^ and sensitized the radiosensitivity of HCC cells by blocking the NF-kB pathway^20^. According to Wu J *et al*.^21^, SFN also inhibits TGF-β-induced epithelial-mesenchymal transition of hepatocellular carcinoma cells via the reactive oxygen species-dependent pathway. A randomized, placebo-controlled trial showed that the disposition of aflatoxin and phenanthrene, both of which could lead to high risk of HCC, was altered by the administration of glucosinolate-rich broccoli sprout preparations^22^.

Significantly, the effect of SFN on HCC angiogenesis has not been reported. We hypothesized that SFN would not only affect EC function but also the interaction between HCC cells and ECs, which would result in the suppression of tumor growth. To test this hypothesis, human umbilical vein endothelial cells (HUVEC) and human hepatocellular carcinoma cells (HepG2) were employed to investigate the anti-angiogenic potentials of SFN *in vitro*. In addition, *ex vivo* aortic ring and *in vivo* tumor-bearing CAM models were used to confirm the anti-cancer properties of SFN. Our findings indicated that SFN inhibited HepG2-induced angiogenesis and that this was associated with the inhibition of STAT3/HIF-1α/VEGF signalling. Thus, the use of SFN could present a strategy for prevention and treatment of HCC.

## Results

### SFN inhibited cell viability and migration of HUVECs

**Figure 1:**
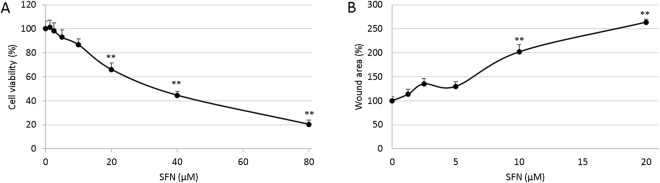
Effect of SFN on cell viability and migration of HUVECs. (**A**) Cell viability at 24 hours SFN treatment was determined by MTT assay. (**B**) Cell migration at 12 hours SFN treatment was measured by wound assay. Data are presented as mean ± SD (n ≥ 5), **p < 0.01 compared to control.

Proliferation and migration are essential characteristics of endothelial cells for the generation of new blood vessels. The MTT assay was employed to evaluate the toxicity of SFN to HUVEC cells. Cells were incubated with different doses of SFN for 24 hours. Results showed SFN inhibited cell viability in a dose-dependent manner (Fig. 1A). As determined with logarithmic regression analyses, the half-maximal inhibitory concentration (IC_50_) of 24 hour SFN treatment was approximately 39.1 µM against HUVEC. In further experiments, 0–20 µM SFN dose was used to avoid strong toxicity effect.

To analyse the effect of SFN on HUVEC cell migration, a wound assay was performed on the confluent monolayers of HUVEC cells. Vehicle control (0.1% DMSO) reformed a confluent monolayer within 24 hours so a 12-hour time point was chosen. SFN was tested across a concentration range of 1.25–20 µM. As showed in Fig. 1B, SFN treatment inhibited HUVEC migration into the wound in a dose-dependent manner shown as the increased wound area compared to control.

### SFN inhibited tube formation of HUVECs in 3D co-culture with pericytes and microvessel sprouting in mouse aortic rings

**Figure 2:**
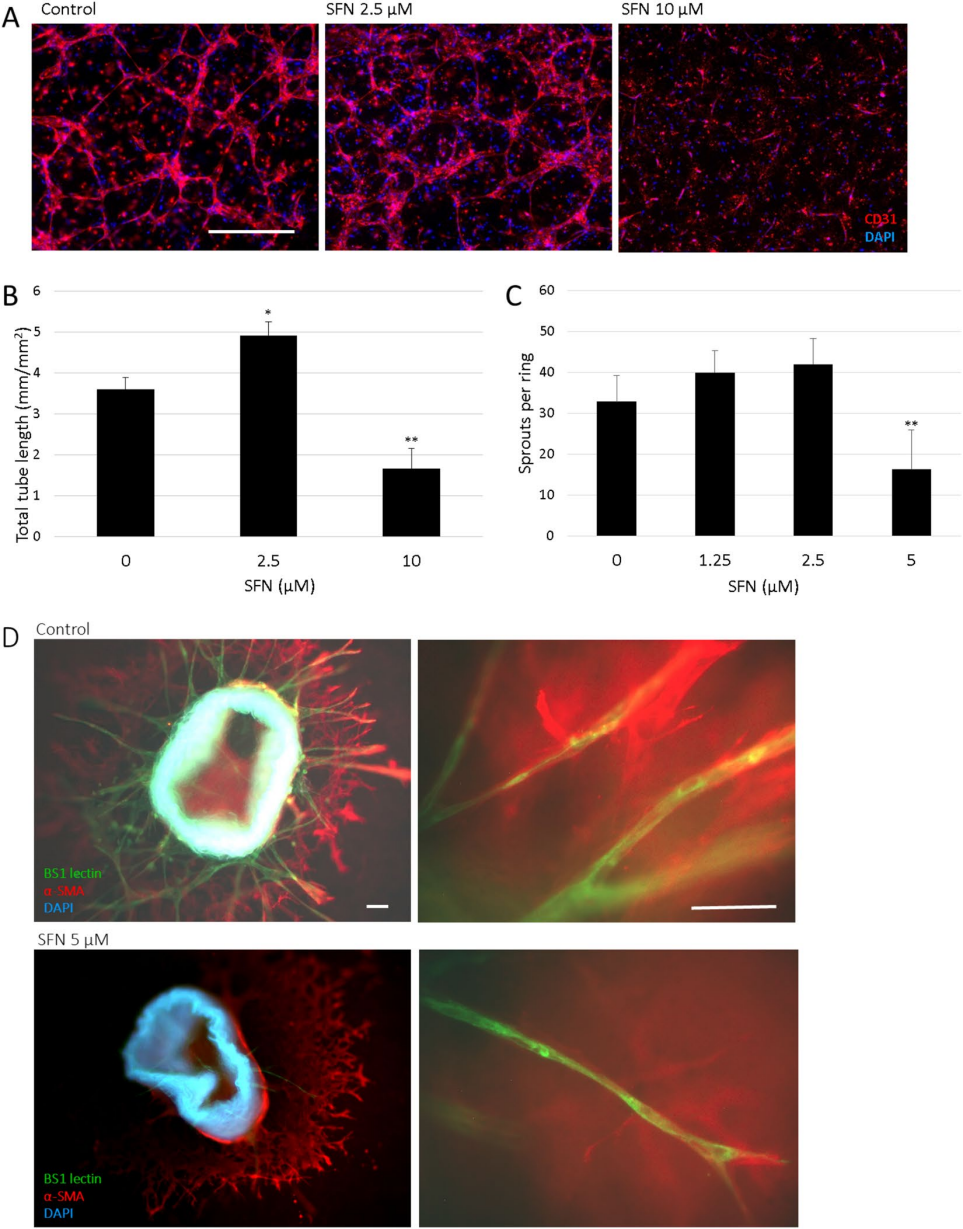
Effect of SFN on tube formation of HUVECs and microvessel sprouting in mouse aortic rings. (**A**) Representative pictures from 3D co-culture of HUVECs and pericytes M2 at day 4 with SFN 0, 2.5, 10 µM. HUVEC were identified by immunodetection of CD31 (red), nuclei were stained (blue) and merged pictures are shown. Scale bar = 500 µm. (**B**) Total lengths of CD31 positive tubes were measured and expressed as mean ± SD (n ≥ 5), *p < 0.05, **p < 0.01 compared to control. (**C**) Dose response of SFN on microvessel sprouting from aortic rings embedded in collagen with DMSO (0.1%) as control. Data are presented as mean ± SD (n ≥ 5), **p < 0.01 compared to control. (**D**) Representative pictures from the immunofluorescent staining of aortic rings. Endothelial sprouts were stained with BS1-lectin-FITC (green), supporting cells were stained for α-smooth muscle actin (red), nuclei were stained with DAPI (blue) and merged pictures are shown. Scale bar = 100 µm.

It has become evident that the establishment of functional capillary networks by ECs, a crucial step for angiogenesis, depends heavily on the interactions and communications between ECs and the surrounding cells. A 3D co-culture model of HUVEC and pericytes M2 was used to study the effect of SFN on HUVEC tube formation (Fig. 2A,B). When cultured in collagen with pericytes, HUVECs form three-dimensional, capillary-like tubular structures. SFN at 10 µM reduced tube formation by 46% compared to control. The destructive effect suggested that SFN could disrupt the formation of enclosed capillary networks of HUVECs.

The effect of SFN on angiogenesis was further explored in a 3D *ex vivo* mouse aortic ring assay (Fig. 2C,D). This model approximates the complexities of angiogenesis *in vivo*, from endothelial cell activation to pericyte acquisition and remodelling. The microvessel sprouting from aortic rings embedded in collagen formed a network of vessels after around 5 days, while 5 µM SFN notably inhibited sprouting of microvessels. Together, these data demonstrated that SFN could inhibit angiogenesis.

### SFN inhibited HepG2-stimulated migration, adhesion and tube formation of HUVEC

Interaction between cancer cells and endothelial cells is implicated in tumor angiogenesis and metastasis^23^. Therefore, the effect of HepG2 cells on HUVEC cell migration, adhesion and tube formation, in addition, the role of SFN in the interaction between HepG2 and HUVEC cells was examined. The cytotoxicity of SFN on HepG2 was assessed (Supplementary Fig. S1) and doses without a significant cytotoxic effect (≤20 µM) were used in all experiments.

**Figure 3:**
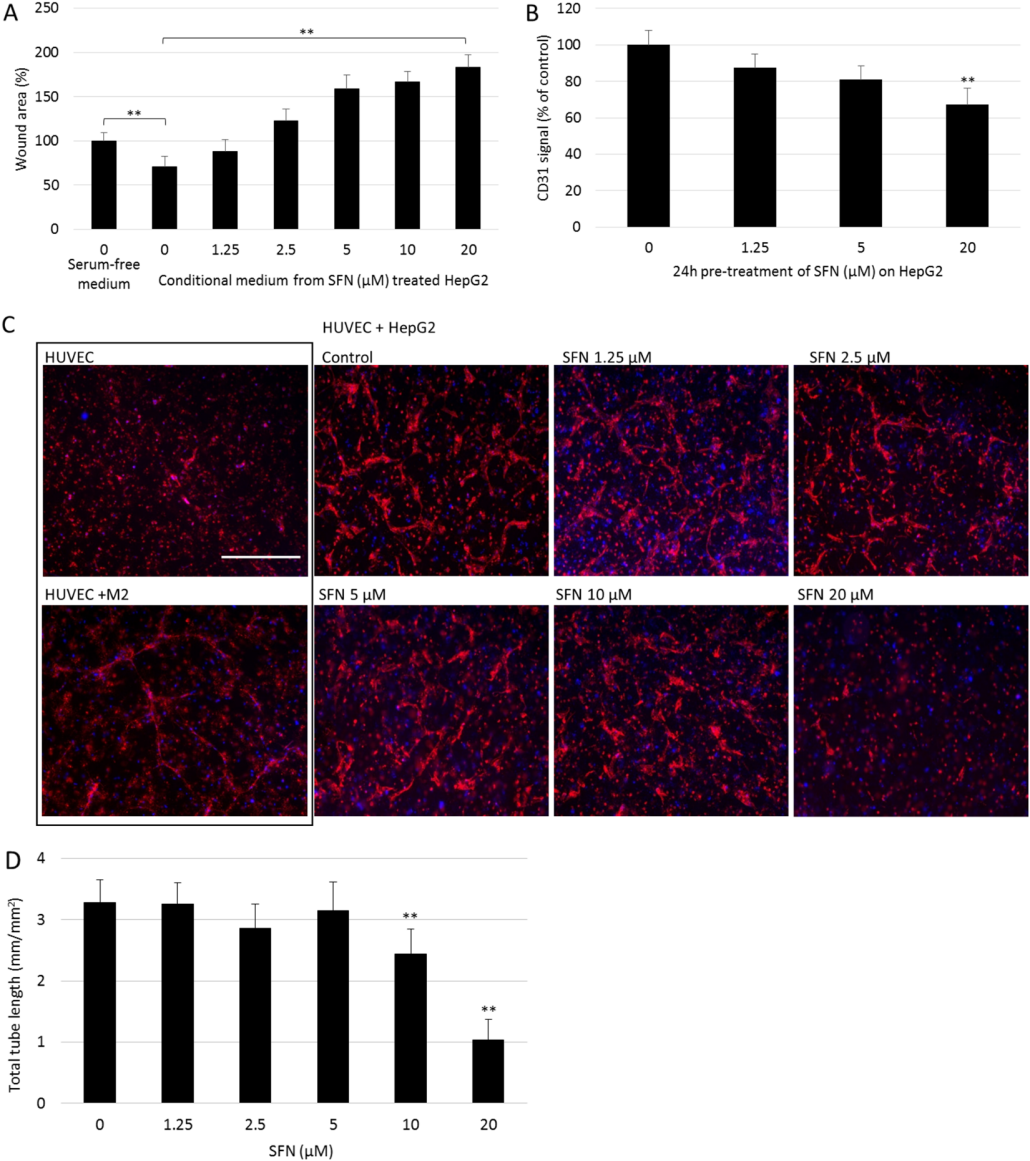
Effect of SFN on HepG2-stimulated migration, adhesion and tube formation of HUVEC. (**A**) CM were collected from SFN treated HepG2 cells then added to HUVECs in the wound assay, with serum-free medium as control. Data are presented as mean ± SD (n ≥ 5), **p < 0.01 between the indicated groups. (**B**) Adhesion assay of HUVECs on 24 hours SFN pre-treated HepG2 cells was performed. Adherent HUVECs were then stained for CD31. Data are presented as mean ± SEM (n ≥ 5), **p < 0.01 compared to control. (**C**) Representative pictures from 3D co-culture of HUVECs and HepG2 at day 3 treated with different doses of SFN, cells were then fixed and stained with CD31 (red) and DAPI (blue). Scale bar = 500 µm. (**D**) Total lengths of CD31 positive tubes were measured and expressed as mean ± SD (n = 5), **p < 0.01 compared to control.

Firstly, the effect of SFN on the potential of HepG2 cells to promote migration of the HUVECs was investigated. HepG2 cells were treated with SFN at different doses for 24 hours, conditional medium (CM) were then collected from DMSO treated groups (control CM) and from SFN (1.25–20 µM) treated groups. These CM were then used in the HUVEC wound assay to determine their effect on HUVEC migration (Fig. 3A). Compared with serum-free controls, the gap area in control CM group was significantly smaller (p < 0.05), indicating that CM from HepG2 cells stimulated HUVEC migration. In addition, this stimulation was suppressed by SFN treatment in a dose-dependent manner, which indicated that SFN treatment could inhibit the pro-migration effect of HepG2 cells on HUVECs.

The ability of HepG2 to recruit endothelial cells was then tested using the cell adhesion assay. Adhesion of HUVECs was tested on the monolayer of HepG2 cells pre-treated by different doses of SFN for 24 hours; CD31 was used for in-well immunoblotting to measure the number of HUVECs. Results showed a dose-dependent decrease of HUVEC cell numbers adhered to SFN pre-treated HepG2 cells (Fig. 3B), which indicated the potential of HepG2 cells to attract HUVEC was significantly compromised by SFN treatment.

Finally, the 3D co-culture tube formation model was modified, using HUVEC and HepG2 cells to investigate the effect of SFN on the formation of vascularization induced by HepG2. Co-culture of HUVECs with pericytes was used as positive control and HUVECs alone as negative control. As shown in Fig. 3C, HUVECs alone did not show any tubular structures after 3 days while co-cultured with pericytes did. More interestingly, HUVEC co-culture with HepG2 displayed scattered distribution of tubular structures indicating HepG2 could induce tube formation of HUVECs but the formed tubes were less mature compared to those induced by pericytes. This might because vessels induced by cancer cells either via angiogenesis or vasculogenesis are usually structurally and functionally abnormal^24^. Treatment with SFN inhibited the formation of the cellular network induced by HepG2, with almost no capillary tubes visible upon 20 µM treatment (Fig. 3D). Overall, these results showed that SFN treatment inhibited the HepG2-induced chemotactic motility and tube formation of endothelial cells.

### SFN inhibited the expression of STAT3, HIF-1α and VEGF in HepG2 cells

**Figure 4:**
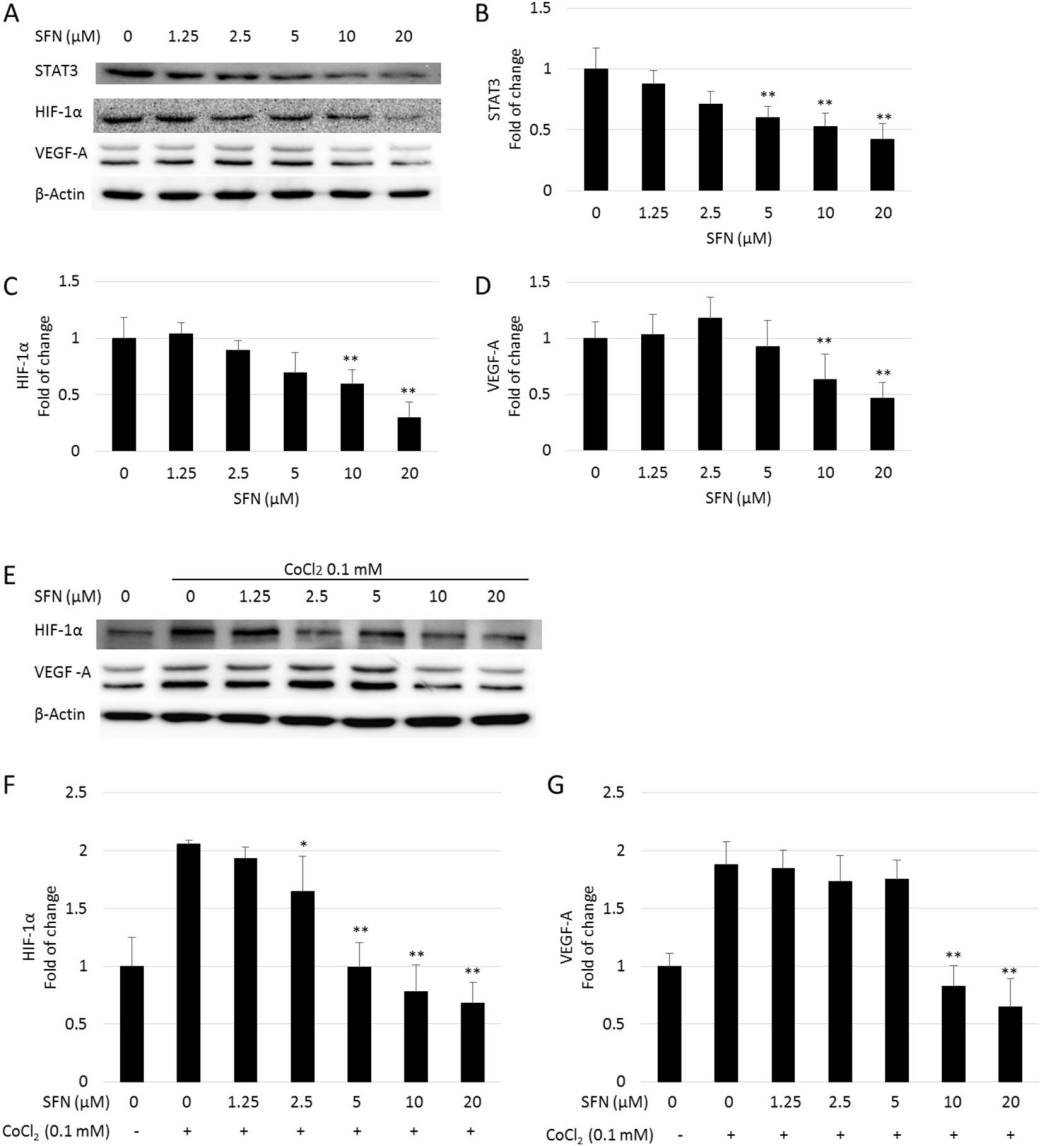
Dose response of SFN on protein expression in HepG2 cells. Cells were treated with 0–20 µM SFN without (**A**) or with 0.1 mM CoCl_2_ (**E**) for 24 hours, whole cell lysates were collected as described and subjected to Western blotting for STAT3, HIF-1α and VEGF-A. β-actin was used as a loading control. Band densities were normalized against β-actin, and results were expressed as fold induction relative to controls (**B**–**D**,**F** and **G**). Data are expressed as means ± SD (n = 3). *p < 0.05, **p < 0.01 compared to control.

The induction of angiogenesis can be mediated by a variety of molecules released by tumor cells. Vascular endothelial growth factor (VEGF) is considered one of the most important angiogenic stimulators, and was identified as a key angiogenic signal in HCC. The expression of VEGF is largely controlled by two major transcription activators: signal transducer and activator of transcription 3 (STAT3) and hypoxia inducible factor-1 alpha (HIF-1α). To further investigate the mechanism behind the inhibition effect of SFN on tumor angiogenesis, its effect on the STAT3/ HIF-1α/VEGF pathway was investigated in HepG2 cells (Fig. 4). Immunoblotting revealed that STAT3 signalling is constitutively activated in HepG2 cells and SFN suppressed the expression of STAT3 in a dose-dependent manner. The effect of SFN on phosphor-STAT3 (Tyr-705) was also investigated, however the reduced p-STAT3 (Tyr705) expression was associated with the reduction of the total STAT3 protein levels (Supplementary Fig. S2). As shown in Supplementary Fig. S3, SFN and two of its metabolites, namely SFN-GSH and SFN-Cys, exhibited similar inhibitory effects on STAT3 in HepG2 cells; and co-treatment with a well-known antioxidant N-acetyl-L-cysteine (NAC) blocked the reduction in STAT3 by SFN and its metabolites, indicating they induced ROS-dependent inhibition of STAT3 signalling. This agreed with previous findings by Miao *et al*.^25^.

SFN at 10 and 20 µM significantly down-regulated the expression of HIF-1α, it also dramatically inhibited cobalt chloride (CoCl_2_)-induced accumulation of HIF-1α in a dose-dependent manner, suggesting SFN inhibited the synthesis of HIF-1α. The protein expression level of VEGF-A was also reduced by SFN with or without CoCl_2_ induction, in accordance with the results of HIF-1α. These results demonstrated that SFN could down-regulate the STAT3/ HIF-1α /VEGF-A pathway in HepG2 cells, and this could be the mechanism behind the inhibitory effect of SFN on HepG2-stimulated migration and tubulogenesis of endothelial cells.

### SFN suppressed tumor growth and angiogenesis in the modified CAM model

**Figure 5:**
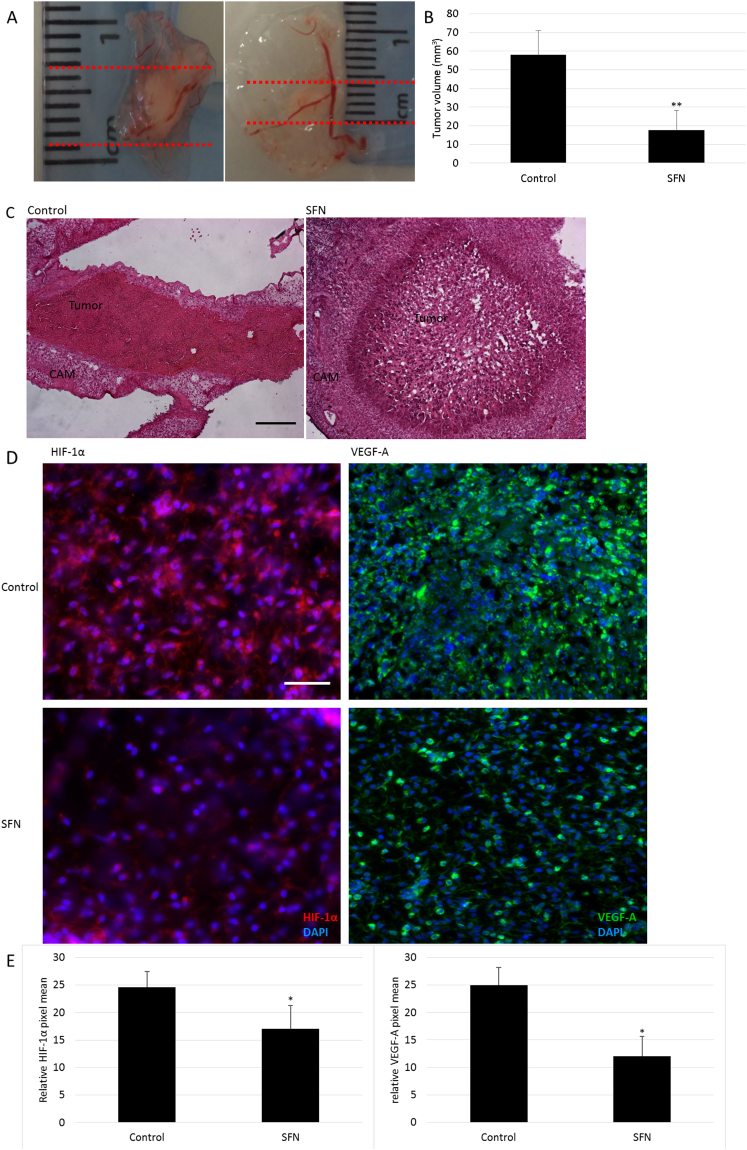
The effect of SFN in the HepG2-bearing CAM model *in vivo*. (**A**) Representative photos of the tumor samples from control (0.1% DMSO) or SFN (20 µM) treatment. (**B**) The tumor volume was determined by direct measurement with callipers and results expressed as mean ± SD (n = 5; **p < 0.01). (**C**) Representative pictures from H&E staining of tumors, scale bar = 200 µm. (**D**) Representative pictures from IHC staining for HIF-1α and VEGF-A of tumors, scale bar = 200 µm. (**E**) HIF-1α and VEGF-A staining intensities (mean ± SD, n ≥ 5) were quantified. *p < 0.05 compared to control.

A modified CAM assay was used to assess the influence of SFN on tumor growth and angiogenic potential of HepG2 cells *in vivo*. HepG2 cells (1 × 10^6^) were mixed with growth factor reduced matrigel containing DMSO (0.1%) or SFN (20 µM), and incubated on top of the chicken embryo chorioallantoic membrane for 3 days. The tumors were then harvested and measured to calculate their volumes. The tumors treated with SFN had a significantly smaller volume compared with the control group (Fig. 5A,B). H&E staining demonstrated massive areas of necrosis within SFN-treated tumors (Fig. 5C). As HepG2 cells were resistant to SFN toxicity, the reduction in tumor size by SFN is less likely to result from its effect on tumor cell population but to its effects on vasculature formation within tumor stroma. The results of IHC analysis showed that SFN reduced the level of HIF-1α and VEGF in the tumor tissues (Fig. 5D,E), which is consistent with the in *vitro* results. Thus, SFN reduced tumor growth and angiogenesis.

## Discussion

HCC is one of the most common human cancers but is associated with poor prognosis. Growth and metastasis of HCC solid tumors depend on angiogenesis, thus preventing or inhibiting angiogenesis by non-toxic, affordable, and effective phytochemicals could be a useful strategy for better management of HCC.

SFN, a predominant dietary phytochemical in cruciferous vegetables, has been studied intensively in terms of cancer prevention and treatment. More recently, its effects on endothelial cell functions have been reported *in vitro* ^26,27^ as well as *in vivo* ^28^. In terms of tumor angiogenesis, SFN has been reported to inhibit NF-κB-regulated VEGF expression in human prostate cancer cells^29^; and hypoxia-induced HIF-1α and VEGF expression in human colon cancer cells^30^. It can also reduce the production of several pro-inflammatory cytokines and pro-angiogenic growth factors in human breast cancer cells^31^; and enhance the therapeutic efficacy of TRAIL in a prostate cancer orthotopic model through regulation of apoptosis, metastasis, and angiogenesis^32^. In the present work, it is demonstrated that SFN not only interfered with endothelial cell proliferation, migration and tube formation, but, for the first time, inhibited the pro-angiogenic effect of HepG2 cells both *in vitro*, *ex vivo* and *in vivo*.

Endothelial cells are one of the critical components in the tumor microenvironment and play a crucial role in the growth and progression of cancer through angiogenesis^33^. Thus the inhibition of their function is explored as a potential therapy. In this study, SFN significantly inhibited HUVEC viability and migration, in agreement with previous results^34,35^. High dose of SFN also disrupted the ability of HUVECs to form capillary-like tubular structure in 3D co-culture with pericytes, and blocked microvessel sprouting from mouse aortic rings *ex vivo*. These two angiogenesis models very closely mimic the physiology of vascular maturation because of the involvement of pericytes and smooth muscle cells^36^. Therefore, the anti-angiogenic effects of high dose SFN (>5 µM) has been confirmed. Interestingly, 2.5 µM SFN simulated tube formation by 37% in the 3D co-culture model and sprouting by 28% in the aortic ring assay. The biphasic response of SFN has also been reported in other studies^37,38^ as a potential risk factor in its application for cancer treatment. Further *in vivo* studies are needed to confirm this hormetic effect. As the physiological concentration of SFN by consumption of a meal rich in cruciferous vegetables or from supplements is around 1–7 µM in human plasma^39,40^, it is crucial to achieve targeted delivery of high dose of SFN for cancer chemoprevention or treatment. Conversely, low dose SFN could be beneficial to patients with cardiovascular disease because of its stimulation effect on endothelial cell tube formation.

Intercellular communication and chemotaxis play key roles in the angiogenic process of HCC and can occur via direct contact or paracrine signalling between tumor cells and host microenvironment such as ECs^33^. However, the crosstalk between tumor cells and ECs is still in need of further investigation. In this study, the influence of HepG2 on HUVEC angiogenic behaviour, and the effect of SFN on the interaction between HepG2 and HUVEC have been studied. Results showed that HepG2 cells stimulated HUVEC migration and tube formation, and that this stimulation could be inhibited by SFN pre-treatment on HepG2 cells. In addition, SFN could interrupt the ability of HepG2 cells to recruit HUVECs, indicating that SFN may influence not only paracrine factors but also cell-cell interactions between HepG2 and HUVEC.

VEGF-A has been implicated as a major paracrine mediator in the pathogenesis of HCC. In clinical trials, targeting VEGF pathways has been effective in treating HCC but is also prone to developing resistance and more aggressive tumors, which could be due to the activation of transcriptional factors by anti-angiogenic agents^41^. Thus, targeting transcriptional factors may be more effective than targeting VEGF and its receptors. STAT3 and HIF-1α, two major transcription factors that regulate VEGF, have been found to be consistently upregulated in various cancers including HCC and associated with poor clinical outcomes in patients^42,43^. Suppression of STAT3 and HIF-1α activity was demonstrated to inhibit the growth in HCC^44^. Here, the inhibition of STAT3 by SFN treatment were verified, which also coincided with the reduced HIF-1α and VEGF expression in HepG2. These data were consistent with previous findings in other cell lines^25,45,46^, indicating the STAT3/ HIF-1α /VEGF may be responsible for the anti-angiogenic effects of SFN.

Under normal conditions, HIF-1α is hydroxylated by prolyl hydroxylases (PHDs) at oxygen-dependent degradation (ODD) domains at proline 402 and 564, and then interacts with the von Hippel-Lindau (VHL)-ubiquitin E3 ligase complex before being degraded by the ubiquitin-proteasome system. Under hypoxia or stimulation with certain growth factors or cytokines, HIF-1α can escape degradation and bind with HIF-1β. The heterodimeric HIF1 formed rapidly translocates to the nucleus and activates hypoxia-responsive elements (HREs) which regulate many genes involved in cancer biology such as angiogenesis, metabolic adaption, cell survival and metastasis^42,47^. CoCl_2_, widely used as a hypoxia mimicking agent, can stabilize HIF-1α by inhibiting PHDs activity^48^ as well as the binding between pVHL and hydroxylated HIF1^49^. HepG2 cells treated with CoCl_2_ were shown here to increase expression of HIF-1α and VEGF, this increase was blocked by co-treatment of SFN in a dose-dependent manner. This indicates SFN could influence the synthesis of HIF-1α. The same result was found in MCF7, 4T1 and 293 cells by Zhou J and coworkers^50^.

The antitumor efficacy was demonstrated *in vivo* by a modified CAM assay, which has been used previously to study tumor angiogenesis, invasion and metastasis in malignancies including HCC^51^. The highly-vascularized nature of the CAM enables the survival of embedded tumor cells and the presence of extracellular matrix proteins in CAM mimics the physiological cancer cell environment. Here tumor volume was reduced by SFN treatment, which was consistent with the widespread tumor necrosis indicated by H&E staining. The expression of angiogenic factors tends to reflect aggressive tumor phenotype^6^, whilst the inhibition of HIF-1α and VEGF expression was confirmed by IHC analysis in the SFN treated tumors. These results suggest that the antitumor activity of SFN may be mediated, at least in part, by inhibition of HIF-1α and subsequent VEGF expression.

In summary, the present study confirmed that SFN not only affects ECs function but also the interaction between HCC cells and ECs by inhibiting STAT3/ HIF-1α /VEGF signalling in the cancer cells, which results in the suppression of angiogenesis induced by HCC cells leading to an anti-tumor effect. Based on these results, SFN has the potential to be considered as an anti-angiogenic agent against HCC and would warrant further *in vivo* investigation.

## Methods

### Reagents

SFN was purchased from Toronto Research Chemicals (Toronto, Canada). Complete protease inhibitors were obtained from Roche Applied Science (West Sussex, UK). Primary antibodies to STAT3 (cat. no. 8019), p-STAT3 (cat. no. 8059), β-actin (cat. no. 7210) and α-smooth muscle actin (cat. no. 53142); HRP-conjugated goat anti-rabbit and rabbit anti-goat IgG were purchased from Santa Cruz Biotechnology (Heidelberg, Germany). Primary antibodies to HIF-1α (cat. no. 2185) and VEGF-A (cat. no. 46154) were purchased from Abcam (Cambridge, UK). Anti-human CD31/PECAM-1 (cat. no. 555444) was purchased from BD Biosciences (Oxford, UK). Secondary antibodies conjugated with Cy3 were purchased from Jackson Immuno Research (PA, USA). All other reagents were purchased from Sigma-Aldrich (Dorset, UK) if not mentioned otherwise.

### Cell culture

HUVECs were obtained from TCS Cellworks (Buckingham, UK) and murine MII perivascular cells (M2) were isolated as previously described^52^. HepG2 cells were purchased from ATCC. HUVECs were cultured in Endothelial Cell Growth Medium 2 (PromoCell, Heidelberg, Germany) supplemented with antibiotics (penicillin (100 U/ml) and streptomycin (100 mg/ml) at 37 °C, 5% (v/v) CO_2_. M2 and HepG2 cells were routinely cultured in Dulbecco’s modified Eagle’s medium (DMEM) supplemented with 10% foetal bovine serum (FBS), 2 mM glutamine, penicillin (100 U/ml) and streptomycin (100 mg/ml) at 37 °C, 5% (v/v) CO_2_. HUVECs were used between passages 5 and 9; and M2 between passages 35 and 40 for all experiments. For these two cell lines, cells were grown in flasks coated with 10 µg/ml type-I collagen (Sigma-Aldrich).

### Cell viability assay

The cell proliferation 3-[4,5-dimethylthiazol-2-yl]-2,5 diphenyl tetrazolium bromide (MTT) assay was employed to determine the toxicity of SFN towards cultured cells. Cells were seeded in 96-well plates in DMEM with 10% FBS at a concentration of 0.5–1.0 × 10^4^ cells in a final volume of 100 μl per well. When cells were at approximately 70–80% confluence, different doses of SFN treatments were added with fresh medium, DMSO (0.1%) used as control. After 24 hours, the medium was removed, 100 μl (5 mg/ml) MTT was added, and incubated at 37 °C for 1 hour to allow the metabolism of MTT. The formazan formed was then re-suspended in 100 μl DMSO per well. The final absorbance was recorded using a microplate reader (BMG Labtech Ltd, Bucks, UK) at a wavelength of 550 nm and a reference wavelength of 650 nm.

### Conditional medium collection

Conditional medium (CM) from HepG2 cells was prepared by seeding HepG2 cells into 10 cm dishes with 10 ml complete cell culture medium and when at 80% confluence were washed with PBS and incubated with serum-free medium and SFN (0–20 µM) for another 24 hours. CM was collected from each dish and filter sterilized (0.2 µm, Minisart, Sigma-Aldrich), then stored at −80 °C for further experiments where serum-free medium was used as a negative control.

### Wound assay

HUVEC cells were seeded in 24-well plates at 2 × 10^5^ cells/ml. After cells reached 100% confluence, scratches were made with a 1 ml pipette tip across the centre of the wells. Detached cells were removed by gently washing the well twice with medium. The wells were then filled with fresh medium with different dose of SFN or conditional medium from HepG2. Each treatment was performed at least in triplicate. Cells were grown for additional 12 hours then washed twice with PBS, fixed with ice-cold methanol for 10 mins, stained with 1% crystal violet for 30 mins. At least 3 pictures were taken within each well of the stained monolayer on an inverted microscope at 5 × magnification. The wound area was quantitatively evaluated using ImageJ^53^. Percentages of wound areas from treated wells were then normalized to the wound area from control wells. At least 10 pictures from each treatment were used for the calculation.

### Tube formation in a 3D co-culture model

HUVEC and M2 or HepG2 cells were co-cultured in collagen type I gel according to 5:1 ratio modified as previously^52^. Different doses of SFN were added to the medium on top of 3-D collagen gel with DMSO (0.1%) as control. Medium was changed every 48 hours and cultures were maintained for 3–5 days. Then the whole-mount immunohistochemistry of 3D collagen cultures was performed with CD31 and counterstained with DAPI. Samples were examined by fluorescence microscopy (Axioplan2, Carl Zeiss, Oberkochen, Germany). In five random fields from each sample, the total lengths of CD31-positive tube-like structures were measured by Volocity 4.0 (Improvision, Coventry, UK). Cumulative tube lengths per area are expressed as mm/mm^2^.

### Aortic ring assay

This assay was performed according to the protocol of Baker and Robinson^54^. In summary, thoracic aortae were dissected from mixed background mice, cut into rings approximately 0.5 mm in width and incubated in serum-free Opti-MEM (Gibco, cat. no. 51985026) at 37 °C overnight. Each ring was then embedded in separate wells of a 96-well plate containing 1.2 mg/ml of collagen I (Millipore, cat. no. 08115), which was polymerised by leaving the plate at 37 °C for 30 mins. Rings were fed with fresh Opti-MEM supplemented with 2.5% FBS and VEGF (30 ng/ml) at 37 °C every 3 days with different doses of SFN. After 6 days, rings were fixed with 4% (v/v) formalin and the microvessel counted by phase-contrast microscopy. To further staining, specific epitopes can be fluorescently labelled. After the fixing, the rings were permeabilised with 0.25% (v/v) Triton X-100 in PBS, and blocked with 2% (v/v) BSA in PBLEC (PBS with 1 mM CaCl_2_, 1 mM MgCl_2_, 0.1 mM MnCl_2_, 1% Tween-20) for 30 mins at 37 °C. FITC-conjugated BS-1 lectin (Sigma, cat. no. L9381/L5264) was used to stain endothelial cells and anti-actin α-smooth muscle Cy3 (α-SMA; Sigma, cat. no. C6198) was used to stain the supporting cells. 1 µg/ml DAPI was used to stain the nuclei. Images were taken by Axiovert 40 CFL inverted microscope (Carl Zeiss).

### Adhesion assay

Adhesion between tumor cells and endothelial cells was measured as described with modifications^55^. HepG2 were seeded in 96-well plates to 100% confluence then treated with 0–20 µM SFN for 24 hours. HUVECs was seeded on the HepG2-coated plate at a density of 5 × 10^4^ cells/well in serum-free medium (12 replicates for each treatment). The plate was then incubated at 37 °C, 5% CO_2_ for 1.5 hours, after which unattached cells were washed three times with PBS. 50 μL of 4% PFA was added to each well for 10 mins to fix the adherent cells. An in well Western blotting for CD31 was then performed to measure the adherent endothelial cells. The signal was detected by Odyssey (LI-COR Biosciences, Cambridge, UK) and results were expressed as CD31 signal intensity (% of control). Three independent assays were conducted per experimental design.

### Protein extraction and Western blot analysis

For total protein, cells were washed twice with ice-cold PBS, incubated in 20 mM Tris-HCl (pH 8), 150 mM NaCl, 2 mM EDTA, 10% glycerol, 1% Nonidet P40 (NP-40) containing complete proteinase inhibitor for 30 mins at 4 °C and then harvested and centrifuged at 13,600 g for 15 mins at 4 °C. Supernatant was collected and the protein concentration determined by the Brilliant Blue G dye-binding assay of Bradford using bovine serum albumin as a standard. For the nuclear protein, the extraction was performed using a Nuclear Extract Kit (Active Motif, La Hulpe, Belgium), following the manufacturer’s instructions.

Protein extracts were heated at 95 °C for 5 mins in loading buffer and loaded onto 10% SDS-polyacrylamide gels together with a molecular weight marker. After routine electrophoresis and transfer, the PVDF membrane was blocked with 5% fat free milk in PBST (0.01% Tween 20) for 1 hour and incubated with a specific primary antibody overnight at 4 °C. The membrane was washed three times for 5 mins with PBST and then incubated with the secondary antibody for 1 hour. After further washing, antibody binding was determined by a chemiluminescence detection kit (Amersham, GE Healthcare) and densitometry was measured by Fluor Chem Imager (Alpha Innotech, Devon, UK).

### HepG2-bearing Chick embryo chorioallantoic membrane (CAM) assay

The growth and angiogenic characteristics of HepG2 cells were tested *in vivo* using a modified CAM assay as previously described^56^. *Gallus gallus* White leghorn fertilized eggs were obtained from a commercial breeder, Henry Stuart (Lincolnshire, UK). Eggs were stored at 16 °C then incubated at 37 °C for 9 days. A small window was made in the shell overlying the most vascularized area of each viable embryo and HepG2 cells (1 × 10^6^) mixed with growth factor reduced matrigel (8.9 mg/mL, BD Biosciences) in a total volume of 25 μL with DMSO (0.1%) or SFN (20 µM) was loaded on the top of the CAM (n ≥ 6 CAMs per treatment). The window was resealed with adhesive tape and eggs were returned to the incubator for 3 days. Tumor samples were cut out from the membrane and their sizes were monitored with callipers, the tumor volume (V, mm^3^) was calculated as (L × W × D), where L = length (mm), W = width (mm) and D = depth (mm).

### H&E staining

Histological assessment was made to assess the development of the implanted tumor. Tumor samples from the modified CAM assay were fixed with 4% paraformaldehyde overnight at 4 °C then changed to cryopreservative medium (15% sucrose in PBS) overnight at 4 °C. Tumors were then embedded in 8% gelatin (15% sucrose in PBS) and snap-frozen for -80 °C storage. Serial sections (10 μm) were stained with hematoxylin and eosin. Sections were imaged under light microscope at 5x magnification.

### Immunohistochemical staining

Briefly, section slides were blocked with 5% goat serum in PBS for 30 mins for nonspecific binding, and then incubated with a rabbit monoclonal antibody against human HIF-1α or a mouse monoclonal antibody against human VEGF-A overnight at 4 °C (each at 1:100 dilution in PBS). Slides were washed with PBS and incubated with the goat derived second antibody for 30 mins, then sections were mounted with fluoromount G with DAPI, and examined by fluorescence microscopy. The expression levels of HIF-1α and VEGF-A were assessed by measuring average pixel intensity per unit area of tumor in 5 random microscopic (400x) field in each slice using imageJ software.

### Statistical analyses

Data are represented as the mean ± SD or SEM. The differences between the groups were examined using the one-way ANOVA test, or Student’s t-test. A p value < 0.05 was considered statistically significant.

### Statements of approval and accordance

All animal experiments were performed in accordance with UK Home Office regulations and the European Legal Framework for the Protection of Animals used for Scientific Purposes (European Directive 86/609/EEC).

## Electronic supplementary material

Supplementary Information
