# Supplementary material for: Sulforaphane exerts anti-angiogenesis effects against hepatocellular carcinoma through inhibition of STAT3/HIF-1α/VEGF signalling
Source: Sci Rep. 2017 Oct 4;7:12651. doi: 10.1038/s41598-017-12855-w (PMC5627255; doi:10.1038/s41598-017-12855-w)
Supplement: Supplementary file 1 — Supplementary Information [file 41598_2017_12855_MOESM1_ESM.pdf]

Title

Sulforaphane exerts anti-angiogenesis effects against hepatocellular carcinoma through inhibition of STAT3/HIF-1α/VEGF signalling

Authors and affiliations:

Peng Liu[1], Samuel J. Atkinson[2], Sophia E. Akbareian[1], Zhigang Zhou[1], Andrea Munsterberg[2], Stephen D. Robinson[2], Yongping Bao[1,*]

1 Norwich Medical School, University of East Anglia, Norwich, Norfolk, United Kingdom.

2 School of Biological Sciences, University of East Anglia, Norwich, Norfolk, United Kingdom.

***Corresponding Author:**

Yongping Bao

E-Mail: Y.Bao@uea.ac.uk

Telephone: +44 (0)1603 591778

Mailing address: Bob Champion Research and Education Building, James Watson Road, University of East Anglia, Norwich Research Park, Norwich, NR4 7UQ

Supplementary Data

1. Effect of SFN on HepG2 cell viability

The MTT assay was employed to evaluate the toxicity of SFN to HepG2 cells. Cells were incubated with 0-160 µM SFN for 24 hours. Results showed SFN inhibited cell viability in a dose-dependent manner. As determined with logarithmic regression analyses, the half-maximal inhibitory concentration (IC50) of 24 hour SFN treatment was approximately 55 µM against HepG2. In further experiments, 0 - 20 µM SFN dose was used to avoid strong toxicity effect.

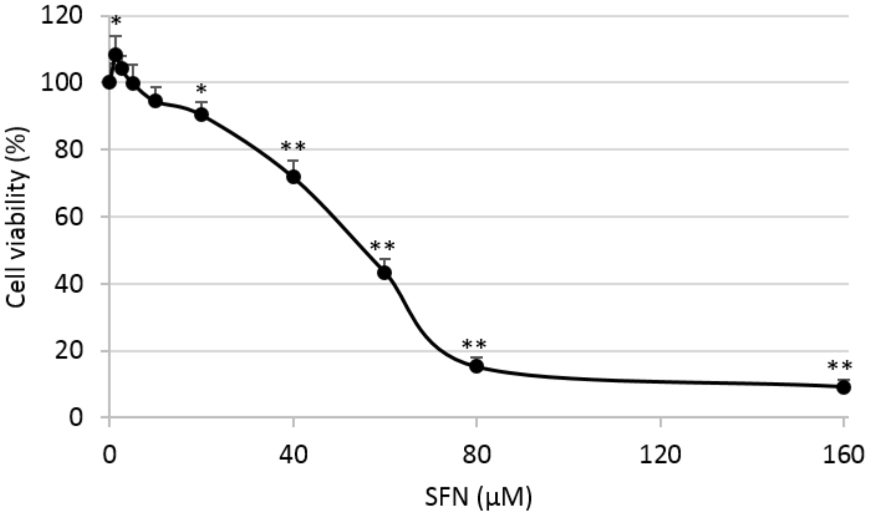

Supplementary Figure 1. Effect of SFN on cell viability of HepG2 cells. Cell viability was determined by MTT assay. Data are presented as mean ± SD (n ≥ 5).

2. Effect of SFN on pSTAT3/STAT3 expression

Immunoblotting was performed to study the effect of SFN on pSTAT3 (Tyr-705) and STAT3 expression in HepG2 cells. To investigate whether the reduced p-STAT3 (Tyr705) expression was due to the reduction of the total protein level, band densities of p-STAT3 (Tyr705) were normalized against STAT3 in the same protein samples. Result showed that the decrease in pSTAT3 was due to the reduction in total STAT3 protein since the ratio of pSTAT3/STAT3 were close to 1, no significant difference between SFN treatment and control (0.1% DMSO).

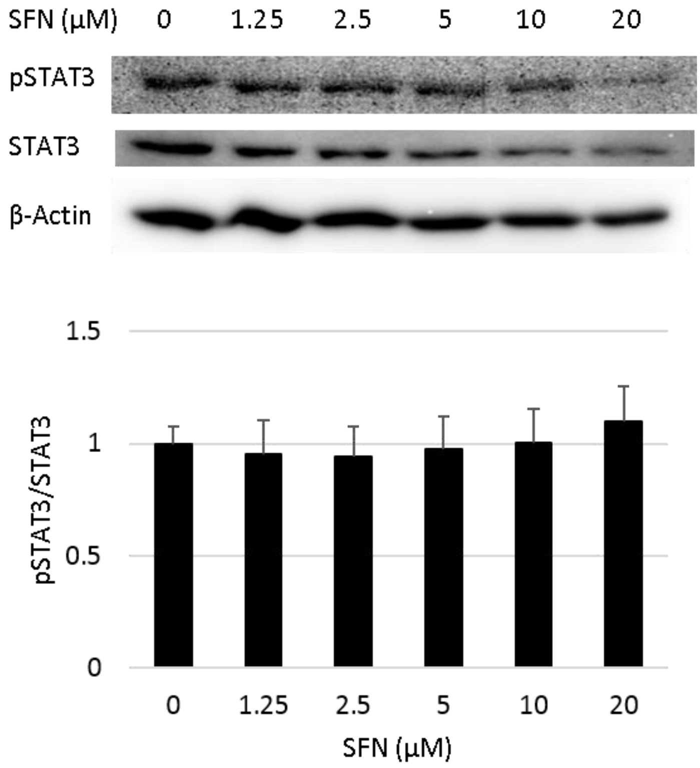

Supplementary Figure 2. Quantification of p-STAT3 (Tyr705) in HepG2 cells under SFN treatment. Experiment carried out as per Figure 4. Band densities of p-STAT3 (Tyr705) were normalized against STAT3, and results were expressed as fold induction relative to controls (means ± SD, n = 3).

3. Effect of SFN and its metabolites on STAT3 expression

To investigate A) whether the bioactivity of SFN is due to its conjugates or to parent SFN released by deconjugation reactions; and B) what is the primary target of SFN (or its metabolites) in the STAT3/ HIF-1α /VEGF signalling pathway, immunoblotting was performed to study the effect of 10 μM SFN and two of its metabolites, namely SFN-GSH and SFN-Cys, on STAT3 expression in HepG2 cells after 24 hours with or without N-acetylcysteine (NAC). Band densities of STAT3 were normalized against β-actin. Result showed that SFN-GSH and SFN-Cys exhibited similar inhibitory effect on STAT3 compared with SFN; and NAC blocked the decrease in STAT3 under treatment of SFN and its metabolites.

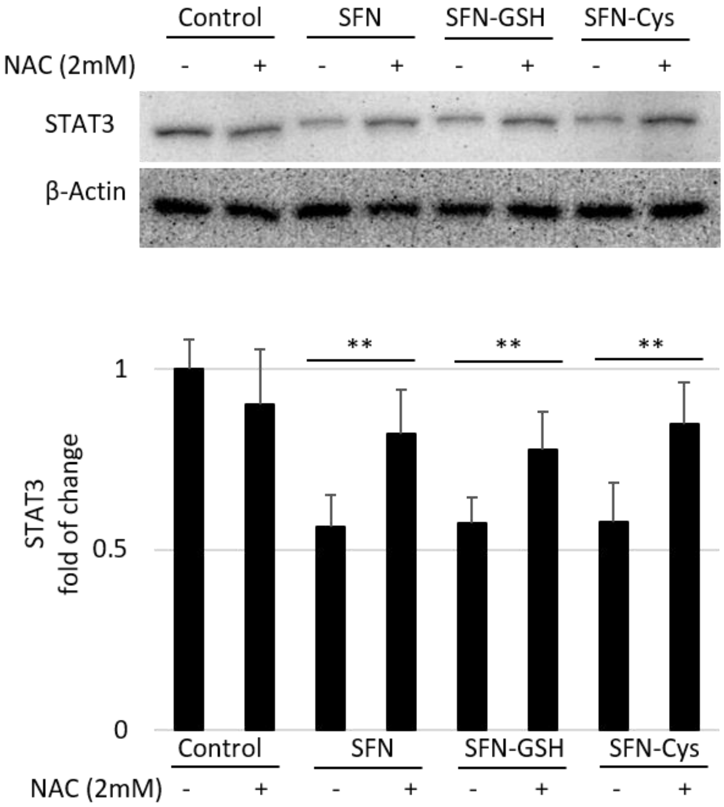

Supplementary Figure 3. Effect of SFN and its metabolites on the expression of STAT3 with or without NAC in HepG2 cells. Cells were treated with DMSO (0.01%), 10 μM SFN, SFN-GSH or SFN-Cys, with or without 2mM NAC for 24 hours. Whole cell lysates were collected and subjected to Western blotting for STAT3. Band densities were normalized against β-actin and expressed as fold induction relative to controls. Data are expressed as means ± SD (n = 3). ** $p < 0.01$ between indicated pairs.
